# Skin disease prevalence study in schoolchildren in rural Côte d'Ivoire: Implications for integration of neglected skin diseases (skin NTDs)

**DOI:** 10.1371/journal.pntd.0006489

**Published:** 2018-05-17

**Authors:** Rie Roselyne Yotsu, Kouamé Kouadio, Bamba Vagamon, Konan N’guessan, Amari Jules Akpa, Aubin Yao, Julien Aké, Rigobert Abbet Abbet, Barbine Tchamba Agbor Agbor, Roger Bedimo, Norihisa Ishii, L. Claire Fuller, Roderick Hay, Oriol Mitjà, Henning Drechsler, Kingsley Asiedu

**Affiliations:** 1 Department of Dermatology, National Center for Global Health and Medicine, Tokyo, Japan; 2 Department of Dermatology, National Suruga Sanatorium, Shizuoka, Japan; 3 Eco Epidemiology Unit, Pasteur Institute Côte d’Ivoire, Abidjan, Côte d’Ivoire; 4 Raoul Follereau Institute Côte d’Ivoire, Adzopé, Côte d’Ivoire; 5 MAP International West Africa, Abidjan, Côte d’Ivoire; 6 National Program for Leprosy Control (PNEL), Ministry of Health and Public Hygiene, Abidjan, Côte d’Ivoire; 7 Department of Medicine, VA North Texas Healthcare System, Dallas, Texas, United States of America; 8 Division of Infectious Diseases, University of Texas Dallas Southwestern, Dallas, Texas, United States of America; 9 Leprosy Research Center, National Institute of Infectious Diseases, Tokyo, Japan; 10 International Foundation for Dermatology; 11 Dermatology Department, Chelsea and Westminster Hospital, London, United Kingdom; 12 Skin NTD Program, Barcelona Institute for Global Health, Hospital Clinic-University of Barcelona, Barcelona, Spain; 13 Global Buruli Ulcer Initiative, World Health Organization, Geneva, Switzerland; University of California San Diego School of Medicine, UNITED STATES

## Abstract

**Background:**

Early detection of several skin-related neglected tropical diseases (skin NTDs)–including leprosy, Buruli ulcer, yaws, and scabies- may be achieved through school surveys, but such an approach has seldom been tested systematically on a large scale in endemic countries. Additionally, a better understanding of the spectrum of skin diseases and the at-risk populations to be encountered during such surveys is necessary to facilitate the process.

**Methods:**

We performed a school skin survey for selected NTDs and the spectrum of skin diseases, among primary schoolchildren aged 5 to 15 in Côte d’Ivoire, West Africa. This 2-phase survey took place in 49 schools from 16 villages in the Adzopé health district from November 2015 to January 2016. The first phase involved a rapid visual examination of the skin by local community healthcare workers (village nurses) to identify any skin abnormality. In a second phase, a specialized medical team including dermatologists performed a total skin examination of all screened students with any skin lesion and provided treatment where necessary.

**Results:**

Of a total of 13,019 children, 3,504 screened positive for skin lesions and were listed for the next stage examination. The medical team examined 1,138 of these children. The overall prevalence of skin diseases was 25.6% (95% CI: 24.3–26.9%). The predominant diagnoses were fungal infections (n = 858, prevalence: 22.3%), followed by inflammatory skin diseases (n = 265, prevalence: 6.9%). Skin diseases were more common in boys and in children living along the main road with heavy traffic. One case of multi-bacillary type leprosy was detected early, along with 36 cases of scabies. Our survey was met with very good community acceptance.

**Conclusion:**

We carried out the first large-scale integrated, two-phase pediatric multi-skin NTD survey in rural Côte d’Ivoire, effectively reaching a large population. We found a high prevalence of skin diseases in children, but only limited number of skin NTDs. With the lessons learned, we plan to expand the project to a wider area to further explore its potential to better integrate skin NTD screening in the public health agenda.

## Author summary

Integration of neglected tropical diseases (NTDs) into the public health agenda has been a priority in global health for the last decade. A common feature shared by several NTDs is skin involvement. Conditions within this group of NTDs have now been classified as skin NTDs to promote wider NTD integration. Several skin NTDs including leprosy, Buruli ulcer, yaws, and scabies are co-endemic in Côte d’Ivoire, West Africa. As children are vulnerable to these diseases, we carried out the first large-scale integrated, multi-skin NTD school survey in a rural district of this country. Our strategy of involving community healthcare workers and dermatologists effectively reached a large population. However, the detection of skin NTDs may have been limited because of the low schooling and attendance rate. We found a high prevalence of skin diseases among schoolchildren (26%), possibly due to poor socio-economic status and air pollution, which requires more attention. This high prevalence of skin diseases posed a challenge for our project as the need for medications and those with dermatological skills exceeded our capacity to reach our initial target population. Our study provides important lessons that will aid the framing of future school skin surveys in sub-Saharan Africa.

## Introduction

Integration of neglected tropical diseases (NTDs) into the public health agenda has been a priority in global health for the last decade [1, 2]. Some NTDs not only share the same geographical distribution, but also skin manifestations such as nodules, patches, ulcerations, and sometimes itchiness as a common feature. This set of NTDs has now been classified as skin NTDs to promote focus and enhance wider NTD integration [3–5]. The fact that they can be detected by visual examination is important in resource-limited settings. Detection and often also diagnosis by community healthcare workers with basic training can potentially increase patient’s access to health-care and enable early treatment. In addition, there is no currently available international guidelines or recommendations for the management except for a few such as leprosy, Buruli ulcer (BU), and yaws, which further justifies this new approach.

Children are particularly at risk from several skin NTDs including leprosy [6], BU [7, 8], and yaws [9, 10]. Early detection of these diseases during childhood could significantly impact the lives of those affected through preventing life-long disabilities and disfigurement [6–10]. This will have a major impact on the futures of these vulnerable children for instance through increasing their opportunity for education. However, the high dependency on traditional healing practices and the low numbers of healthworkers with any training in skin disease in the affected areas often hinder early detection [9, 11–16]. Scabies, which has recently been added to the World Health Organization (WHO) group of NTDs, presents with severe itching [17]. The complications of scabies in a patient extend well beyond disturbances in daily life due to itching, and lead to secondary bacterial skin infection which may cause additional, potentially life-threatening, complications such as nephritis and septicaemia [18, 19]. High transmission rates within families or communities is also a problem in scabies which increases the importance of early detection.

Schools are often used as platforms for disease control interventions as they provide direct access to the target population and are convenient for reaching out to a larger population through education or sensitization. We used a similar approach for skin NTD integration. While school skin surveys for leprosy have proved effective in previous studies, for example in Japan and Brazil [20, 21], in sub-Saharan African countries, this approach has never been tested on a larger scale. Due to the high prevalence of skin diseases among children and the lack of relevant expertise in rural Africa, only a few smaller studies exist [22, 23].

Several skin NTDs, including leprosy, BU, yaws, and scabies are co-endemic in Côte d’Ivoire. Of note, the country reports the highest case number of BU, or *Mycobacterium ulcerans* infection, to WHO with over 1000 cases per year [8]. School skin surveys may be one way to promote integration of skin NTD detection and control/intervention in Côte d’Ivoire. Studies from neighboring countries report a high prevalence of skin diseases among children [16–21], but the spectrum of skin diseases that would be encountered in Côte d’Ivoire is unknown.

Community healthcare workers are an important component of the health system in sub-Saharan African countries, and they cover various health issues within the community. Disease control interventions including HIV/AIDS, expanded program on immunization (EPI), and maternal and child health (MCH) have effectively utilized them in the previous years [24–26]. They have also been involved in leprosy and BU control achieving effective outcomes in some places [27, 28]. Using community healthcare workers in school skin surveys thus has the potential to mitigate the lack of experts currently available in Côte d’Ivoire.

In this study, we aimed to assess the feasibility of implementing school skin surveys using a two-step approach involving screening by community healthcare workers (village nurses), followed by a total skin examination of selected cases by dermatologists. We also aimed to determine the prevalence and spectrum of skin diseases among schoolchildren in a rural area of Côte d’Ivoire.

## Materials and methods

### Study setting

The study took place in Adzopé, a densely populated health district in southern Côte d’Ivoire (285,271 inhabitants in 2015); situated approximately 100 kilometers from the country’s economic capital Abidjan. Geographically, the area consists of multiple hills and rivers covered with tropical rainforest. The region is home to the Raoul Follereau Institute Côte d’Ivoire, which was founded as a hospital for leprosy in 1942, and now also serves as a dermatology tertiary hospital. According to the National Leprosy Program, the district had 53 new cases of leprosy in 2012, and 23 cases in both 2013 and 2014, rendering it among the top five endemic sites in the country. District data on new cases of BU was not collected before the time of our study. Therefore, no data was available despite the presence of known cases of BU in the district (field communication and observation).

**Fig 1:**
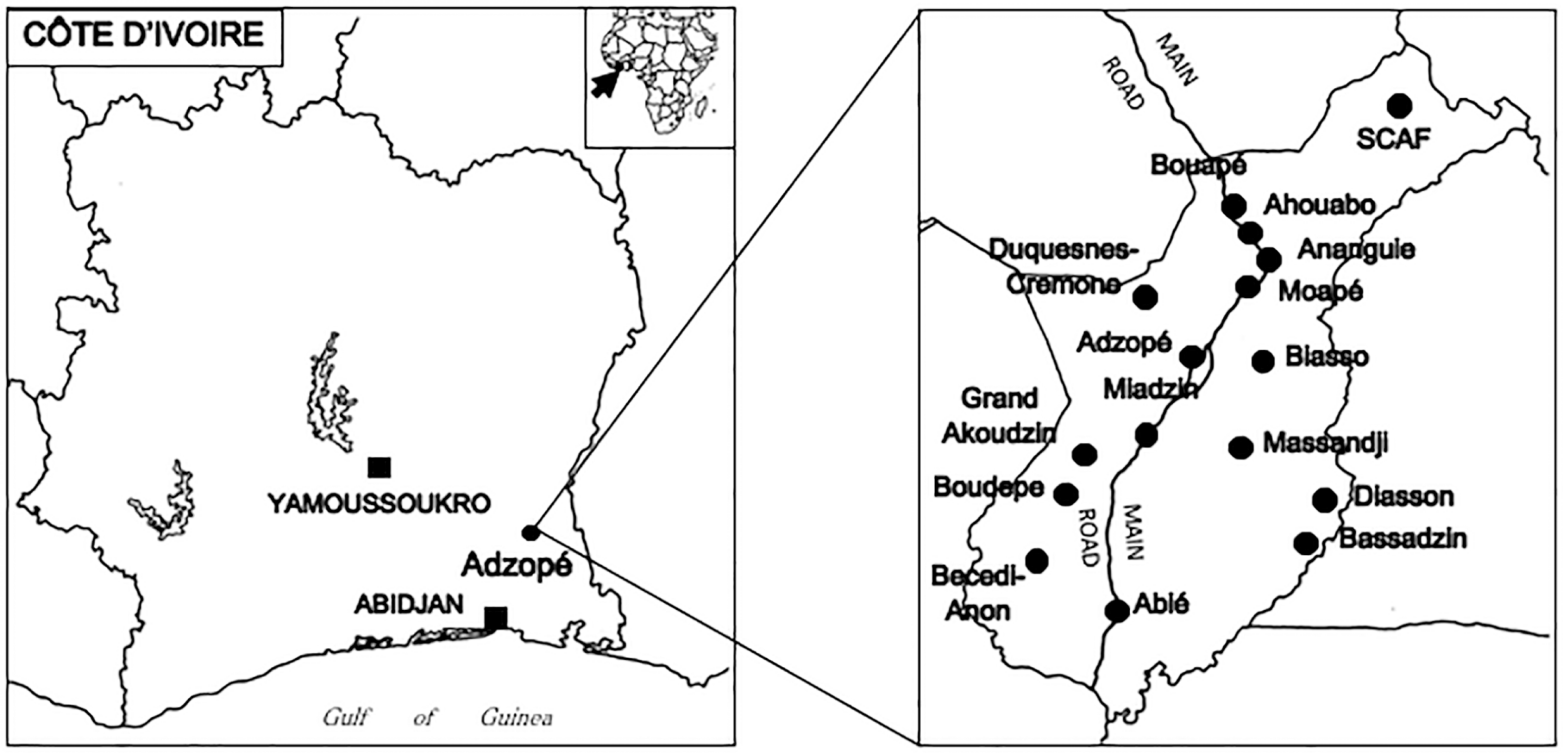
Study sites in Adzopé, Côte d’Ivoire.

We selected the primary schools from 16 villages covering 14,520 children (Fig 1), representing 33% of the total of 39,691 schoolchildren enrolled in the district during the study period. We used non-probability sampling for the selection of villages based on the following two criteria: 1) the district was divided into five zones, we used a quota sampling to ensure representation of subjects by which at least one village from each zone was selected; and 2) we used purposive sampling to maximize the probability of detecting leprosy by which a village was selected if at least one active case of leprosy had been reported there during 2012 to 2014.

### Study procedures

Our mass-screening approach had two phases. In a first phase which took place during November 2015, two village nurses examined the skin, including the scalp, of all primary schoolchildren aged 5 to 15 to identify any skin abnormality. If nothing suspicious was detected the result of the screening was recorded as negative. If they identified any skin lesion, the child’s name was listed for diagnosis and treatment during phase 2 of the study. The first phase was conducted in an integrated manner with another public health program of mass drug administration for soil-transmitted helminthiasis, during which a single dose of albendazole (400mg) was offered to all screened children.

The second phase was conducted between November 2015 and January 2016, and consisted of a total skin examination by a specialized medical team visiting the primary schools. It consisted of 2 dermatologists, 1 physician researcher, 1 school health physician, 4 laboratory technicians, 1 district leprosy coordinator, 2 logisticians and the 2 village nurses who had conducted phase 1. We individually examined each child in an examining space that was set up in an empty classroom. When the light inside the room was dim, we used an outside place with sufficient sunlight where privacy could be ensured. Clinical examination of the entire skin from the scalp to the toes was performed by either one of the two dermatologists in the team. Difficult cases were discussed by both dermatologists and, if necessary, laboratory tests were performed. Diagnosis was mostly based on clinical examination.

In this phase, we provided treatment for all skin diseases other than tinea capitis. We have excluded providing treatment for tinea capitis due to the long treatment duration of this condition: at least 6–8 weeks of griseofulvin. As nature of our study was a survey targeting a large population, we were unable to ensure adherence to treatment with sufficient parent education as well as taking responsive and responsible actions at times of side effects, and thus this decision was made [29]. However, we provided treatment for those with severe forms of tinea capitis, *e*.*g*., extensive lesions or with inflammation.

**Fig 2:**
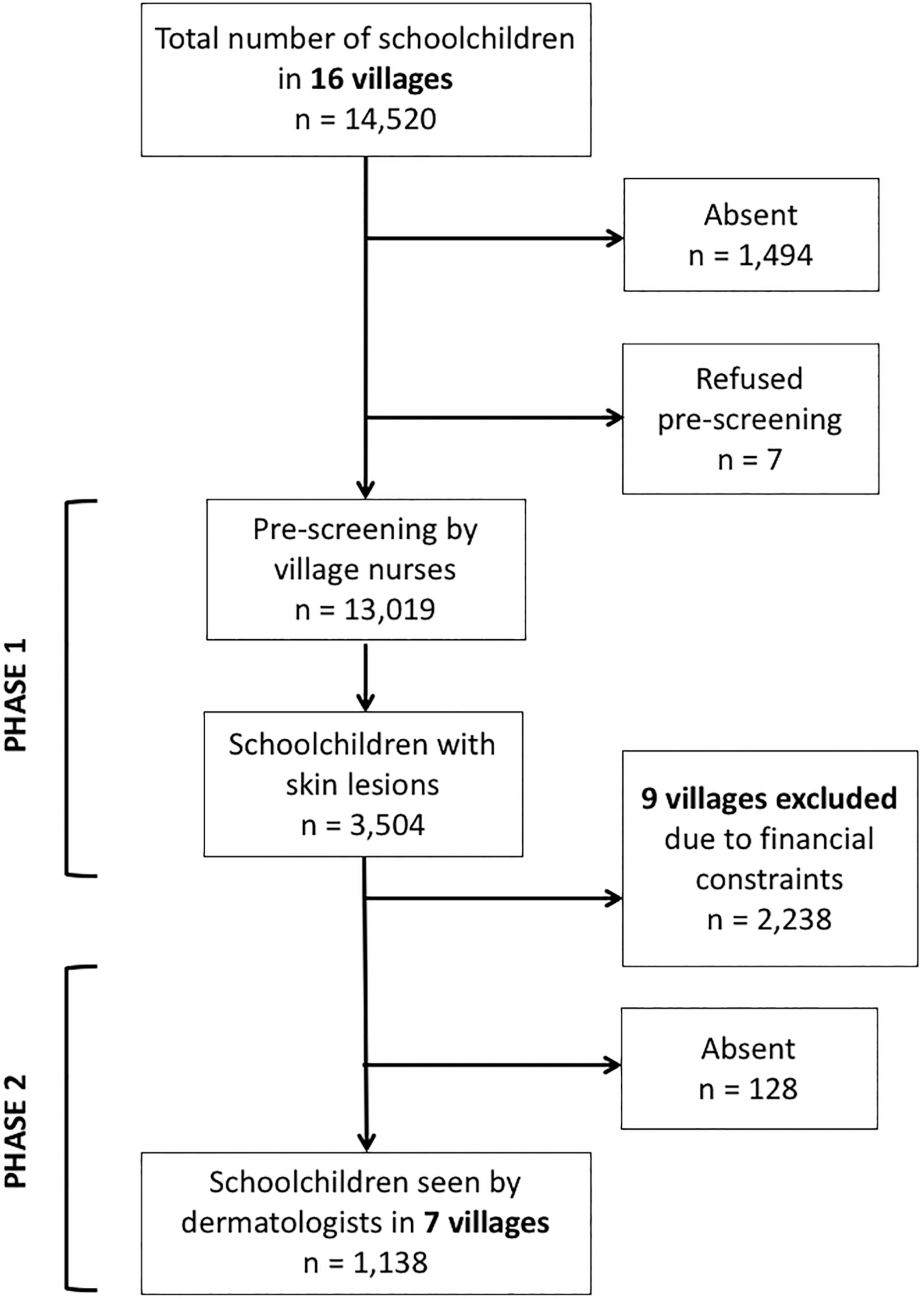
Flow-chart of the school-based skin survey in Adzopé, Côte d’Ivoire.

Due to the unexpectedly large number of children in need of provided dermatological treatment however, the children from 9/16 villages could not be examined in phase 2 as originally planned (see flowchart provided in Fig 2). Thus our findings are based solely on the schoolchildren from the remaining 7 villages (Moapé, Abié, Becedi-Anon, Dequesne Cremone, Boudepe, Massandji, and Diasson) which the medical team visited during phase 2 of the study.

### Data collection and statistical analysis

Data were collected on specially designed forms during the study and then compiled and analyzed using Microsoft Excel 2016 and SPSS version 23. We grouped skin diseases in analogy to a previously reported study [30] and tabulated the number of different types of skin diseases diagnosed in children and the prevalence among the population which was reached by our medical team during phase 2. For diagnoses with a prevalence > 5%, we compared differences in prevalence by gender, age group (5–9 versus 10–15 years) and geographic proximity to the main road. The latter connects Abidjan to the northern areas of the country, and has heavy traffic, likely causing air pollution and dust exposure.

We used Chi-square tests with Bonferroni correction for our demographic comparisons. Where significant differences were found, we calculated the risk ratios.

### Ethics statement

Parents were informed of the study and written informed consent was obtained prior to the enrolment (see S1 Informed consent form). This was facilitated by the school teachers from each class. Our study was approved by the ethics committees of the Ministry of Health (Côte d’Ivoire) (S/N^o^ 627) and the National Center for Global Health and Medicine (Japan) (NCGM-G-001665-01).

## Results

In the first phase, we screened 13,019 schoolchildren from 16 villages reaching 90% of the enrolled children (n = 14,520); 1,494 children were missed due to absence and 7 refused the screening by the village nurses. Twenty-seven percent (n = 3,504) of the screened children had skin lesions and were listed for the next stage examination.

The second study phase was restricted to 7 villages totaling 4,483 schoolchildren (see Methods) of which 1,326 (30%) had screened positive for any skin lesion during phase 1 and 1,138 schoolchildren (25%) underwent total skin examination by our medical team. The demographic characteristics of our study population were recorded in 4,339 schoolchildren (Table 1).

**Table 1:** Demographic composition.

	N	%
Gender		
Boys	2,409	56%
Girls	1,930	44%
Age (years)		
5–9 years	2,005	46%
10–15 years	2,334	54%
Residence		
Road	2,092	48%
Off-road	2,247	52%
TOTAL	4,339	100%

**Fig 3:**
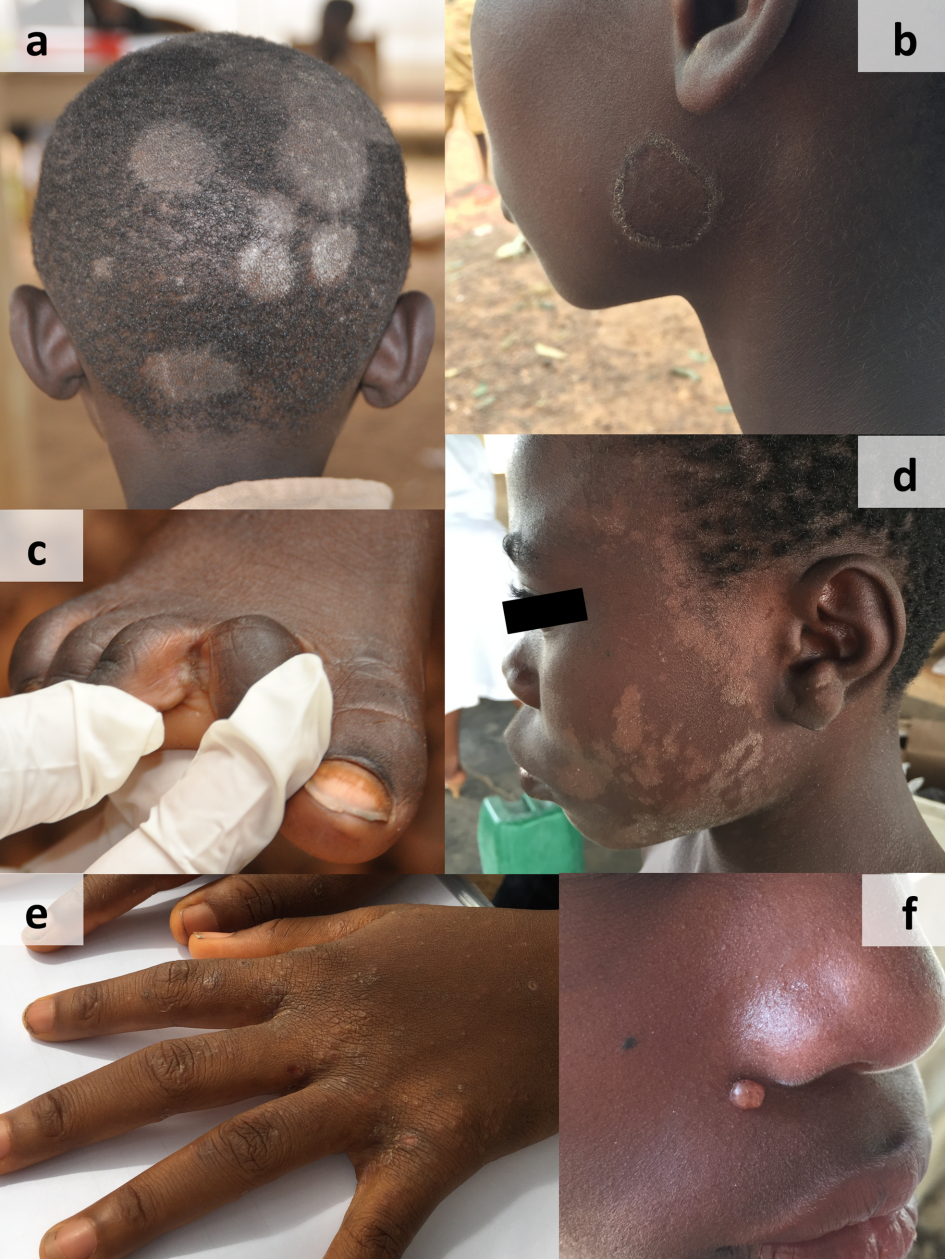
Skin diseases found in schoolchildren in Adzopé, Côte d’Ivoire: (a) tinea capitis, (b) tinea corporis, (c) tinea pedis, (d) pityriasis versicolor, (e) scabies, and (f) molluscum contagiosum.

Table 2 lists the spectrum of skin disease that we encountered. A total of 1,634 skin diseases were diagnosed in a total of 986 children. We identified only 1 case of leprosy and 36 cases of scabies, but a wide spectrum of other skin diseases. Almost 90% of examined children had a skin infection, almost always fungal in nature. More than half of the children had multiple skin diseases. The overall prevalence of skin diseases among schoolchildren in villages that completed the phase 2 intervention was 25.6%, 95% confidence interval (CI): 24.3–26.9%. Fungal infections dominated with a prevalence of 22.3% (CI 21.1–23.5%), followed by inflammatory skin disease (6.9%) and scabies (0.9%). The spectrum of fungal infections was almost entirely comprised of pityriasis versicolor and tinea capitis (11.6% and 11.4% respectively), followed by tinea pedis (6.2%). These conditions can be reliably diagnosed clinically. The causative agents of tinea capitis that were successfully cultured were *Trichophyton (T*.*) mentagrophytes* (n = 30), *T*. *soudanense* (n = 25), and *T*. *rubrum* (n = 1). The prevalence for bacterial and viral infections was very low. Some examples of skin diseases found in children are provided in Fig 3.

**Table 2:** Spectrum of skin diseases diagnosed among schoolchildren in Côte d'Ivoire.

	N	% among diagnosed skin diseases	Prevalence (95% CI)^‡^
Children diagnosed with skin diseases, any type	986	100.0%	25.6 (24.3–26.9)%
Children diagnosed with skin infection	879	89.1%	22.8 (21.6–24.1)%
Children with ≥ 1 mycotic infection			
Total	858 (1,153)*^†^	87.0%	22.3 (21.1–23.5)%
Pityriasis versicolor	447	45.3%	11.6 (10.7–12.6)%
Tinea capitis	437	44.3%	11.4 (10.4–12.3)%
Tinea pedis	237	24.0%	6.2 (5.5–6.9)%
Tinea corporis	28	2.4%	<1.0%
Onychomycose	4	0.9%	<1.0%
Children with ≥ 1 bacterial infection			
Total	22	2.2%	<1.0%
Folliculitis	18	1.8%	<1.0%
Impedigo contagiosum	3	0.3%	<1.0%
Leprosy	1	0.1%	<1.0%
Children with ≥ 1 viral infection			
Total	11	1.1%	<1.0%
Molluscum contagiosum	10	1.0%	<1.0%
Herpes simplex	1	0.1%	<1.0%
Children with ≥ 1 parasitic infection			
Total	36	3.7%	<1.0%
Scabies	36	3.7%	1 (0.7–1.2)%
Children with ≥ 1 inflammatory skin disease			
Total	265 (291)*	26.9%	6.9 (6.1–7.6)%
Seborrheic dermatitis	187	19.0%	4.9 (4.2–5.5)%
Acne vulgaris	37	3.8%	1.0 (0.7–1.2)%
Eczema	25	2.5%	<1.0%
Acute prurigo	11	1.0%	<1.0%
Atopic dermatitis	9	0.9%	<1.0%
Pityriasis rubra pilaire	7	0.7%	<1.0%
Pityriasis rosea	6	0.6%	<1.0%
Prurigo nodularis	6	0.6%	<1.0%
Lichen planus	3	0.3%	<1.0%
Children with ≥ 1 benign skin tumor or nevus			
Total	37 (38)*	3.8%	1.0 (0.7–1.2)%
Congenital nevus	25	2.5%	<1.0%
Café au lait macula	10	1.0%	<1.0%
Cyst	3	0.3%	<1.0%
Children with ≥ 1 miscellaneous skin disease			
Total	81 (83)*	8.2%	2.1 (1.7–2.5)%
Wounds	27	2.7%	<1.0%
Miliaria / heat rash	19	1.9%	<1.0%
Scar	11	1.1%	<1.0%
Vitiligo	10	1.0%	<1.0%
Angular cheilitis	6	0.6%	<1.0%
Localized hyperhidrosis	4	0.4%	<1.0%
Pelagrae	3	0.3%	<1.0%
Alopecia	3	0.3%	<1.0%
No. of skin diseases diagnosed per child ^†^			
1	482	48.9%	
2	352	35.7%	
3	115	11.7%	
4	34	3.4%	
5	3	0.3%	

* Number of children (number of skin diseases)

^†^ 784 had either pityriasis versicolor or tinea capitis.

^‡^ Prevalence among schoolchildren in 7 villages that were reached by the medical team during phase 2. Total number of schoolchildren in 7 villages = 4,483.

**Fig 4:**
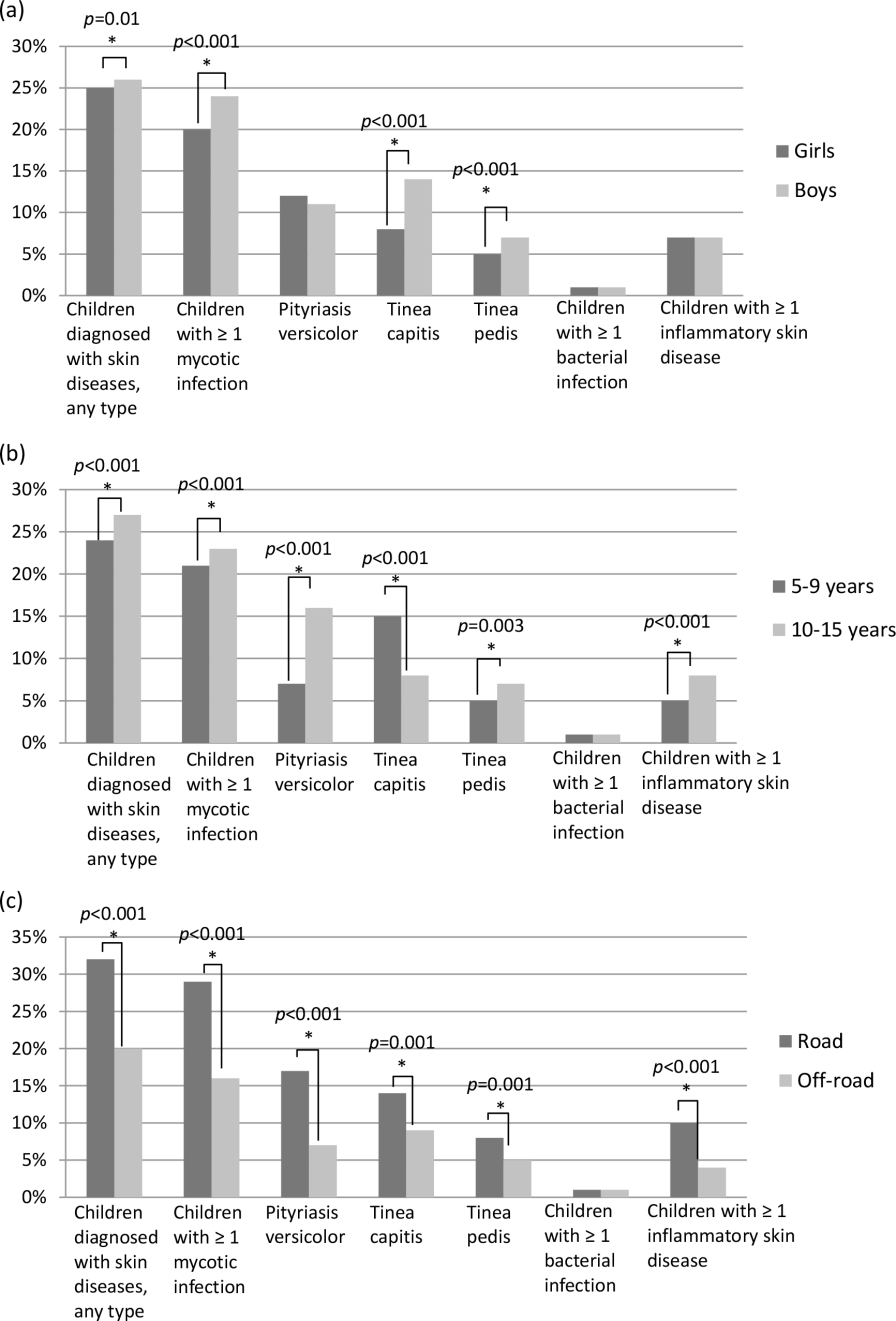
Demographic differences in the prevalence of skin diseases among schoolchildren in Côte d’Ivoire: (a) Gender, (b) Age, and (c) Residence.

Fig 4 depicts demographic differences in the prevalence of skin disease, and Table 3 presents risk ratios for the comparison with significant differences. Boys had a higher prevalence of skin disease than girls, especially for tinea capitis and tinea pedis (14.0% vs. 7.7% and 7.3% vs. 4.6% respectively), notably, almost twice as much than girls. Younger children had higher rates of tinea capitis than teens and preteens (14.8% vs. 7.9%), while the latter had higher rates of pityriasis versicolor (16.0% vs. 7.3%), tinea pedis (6.9% vs. 5.4%), and inflammatory skin diseases (8.3% vs. 5.4%) driven by the higher rates of seborrheic dermatitis and acne vulgaris. Interestingly, children living in villages on the main road had more skin diseases than those off-road (32.2% vs. 19.8%, p<0.001). These children had higher risk of developing both fungal and inflammatory skin diseases, especially the risk was more than twice for pityriasis versicolor and inflammatory skin diseases.

**Table 3:** Risk ratios of skin diseases among schoolchildren according to demographic differences in Côte d'Ivoire.

	Boys vs. girls RR (95%CI)	10–15 yrs vs. 5–9 yrs RR (95%CI)	Road vs. off-road residence RR (95%CI)
Children diagnosed with skin diseases, any type	1.16 (1.10–1.23)	1.28 (1.21–1.36)	1.47 (1.39–1.55)
Children with ≥ 1 mycotic infection	1.26 (1.18–1.34)	1.27 (1.19–1.35)	1.57 (1.48–1.68)
Pityriasis versicolor	—	2.55 (2.31–2.81)	2.24 (2.03–2.46)
Tinea capitis	1.96 (1.77–2.16)	0.62 (0.56–0.68)	1.37 (1.25–1.50)
Tinea pedis	1.73 (1.51–1.98)	1.46 (1.29–1.66)	1.55 (1.36–1.76)
Children with ≥ 1 bacterial infection	—	—	—
Children with ≥ 1 inflammatory skin disease	—	1.77 (1.57–2.00)	2.40 (2.11–2.73)

We had engaged community leaders, school directors/teachers, and other members of the community who all supported us. Schoolchildren’s parents were satisfied with the informed consent before engaging their children in the survey, and there was a very low rate of refusal (0.05%). The study was well received by the local population as children in this area had never before received ‘free skin care’ as described by them and de-worming. Parents were very happy to receive attention for their children’s skin conditions and many accompanied their children to school to see dermatologists in order to receive ‘skin care’ as children also received treatment.

**Fig 5:**
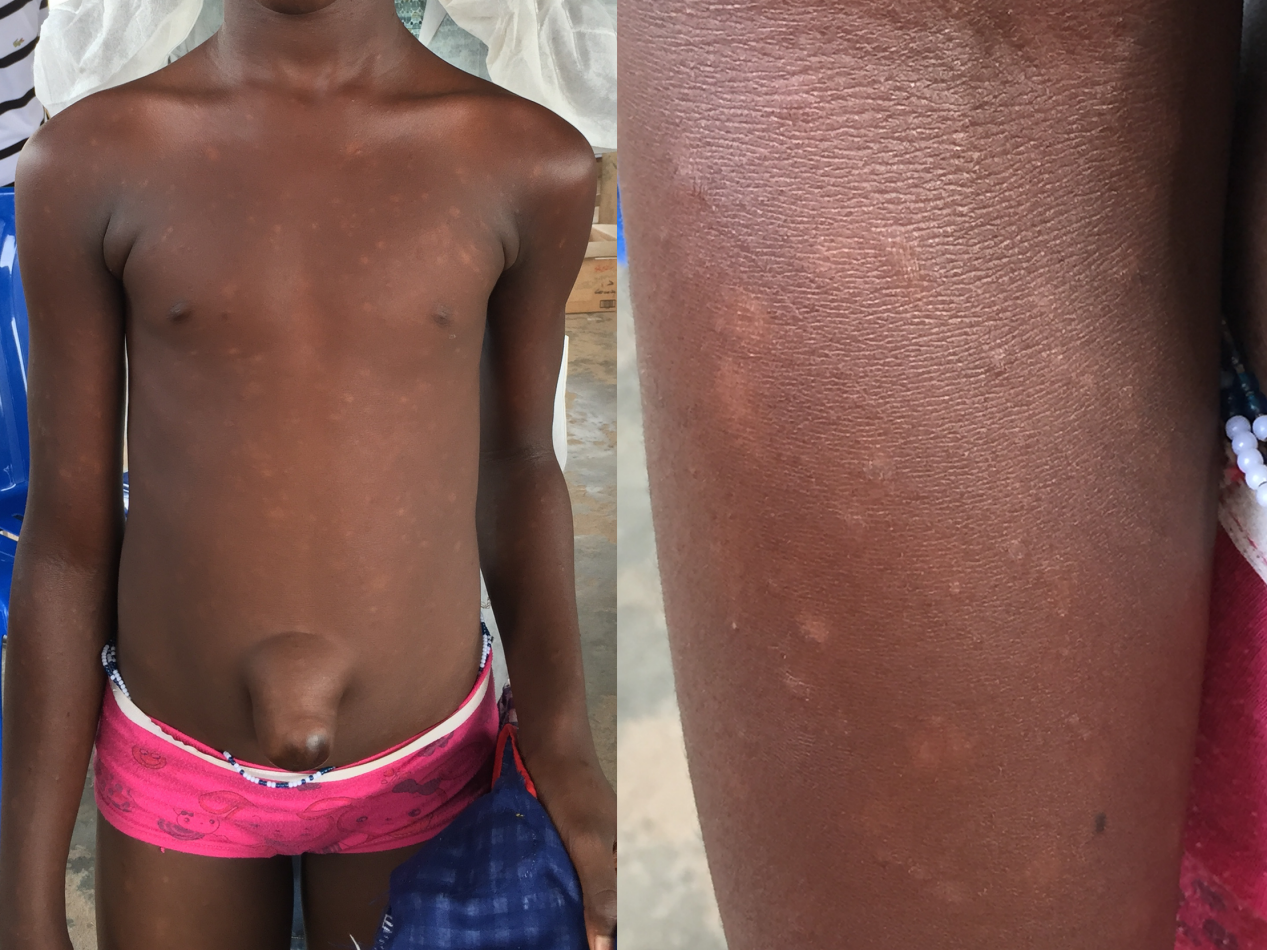
Case of leprosy in a 12-year-old girl detected during the school survey.

One case of lepromatous/ multi-bacillary leprosy was diagnosed in a 12-year-old girl before disability had developed (Fig 5). Her symptoms had started 1.5 years prior to diagnosis. Contact screening was performed but none of her family members was affected.

## Discussion

We used a two-phase strategy to conduct a large-scale school skin survey to increase the yield of detection of any skin NTD as well as a variety of common skin diseases in an area of Côte d’Ivoire known to be co-endemic for skin NTDs. School surveys allow for early detection of disabling or debilitating diseases like leprosy, yaws, and scabies which also prevents spread of the infection in the community and breaks the chain of transmission [21]. In many parts of sub-Saharan Africa where skin NTDs are endemic, there are few dermatologists severely curtailing the prospects for better skin disease control [3, 4]. We assessed the overall burden of skin diseases in children in this rural West-African community, as common skin diseases are equally neglected. We utilized village nurses which allowed us to reach a large number of schoolchildren, thus compensating for the lack of dermatologists. We also combined our skin survey activity with the mass drug administration for soil-transmitted helminthiasis for effective use of opportunities and resources, and likely enhanced community acceptance. Our report is to our knowledge the first of its kind from Africa.

Regardless of our strategy, however, no cases of BU or yaws were diagnosed leaving the total case count for skin NTDs lower than what we expected. One possible explanation for this may be the relatively low schooling rate which is estimated to be only around 75% in Adzopé district [31]. It is possible that our school survey thus missed the most vulnerable population at risk of being affected with infectious diseases–the non-schooling population. In addition, a number of students were absent from school especially during phase 1, possibly due to the long distance from home to school, and also some were helping their parents in the fields. We also could not rule out the possibility that stigma and discrimination might have prevented some children from coming forward although we believe that we largely circumvented this by targeting all skin conditions.

Seasonality might have been also a factor to explain the lack of skin NTD detection in our survey, which took place during the dry season. Yaws is known to be more prevalent during the rainy season [9]. The seasonality of BU is yet unclear, but one recent study has shown a seasonal pattern in the presence of *M*. *ulcerans* in the environment [32]. Therefore, the right choice of season, or ideally a perennial study period is an important consideration for this type of survey.

There was a high prevalence of common skin diseases among schoolchildren (23.3%), which was in concordance with previously published studies from African countries in which prevalence ranged from 26.7 to 80.4% [22, 30, 33–36].

Skin infections, especially fungal ones were by far the most common diagnosis confirming earlier studies [22, 30, 33–36].

In our study, pityriasis versicolor (11.6%) was the most prevalent skin disease. This was followed by tinea capitis (11.3%), which was in line with a previous report from Côte d’Ivoire (13.9%) [37]. Tinea capitis has been reported to be the most common skin disease among schoolchildren in other studies in sub-Saharan Africa but its prevalence varies considerably [22, 30, 36]. Many previous studies reported either low prevalence for pityriasis versicolor, or did not include this condition as a diagnosis, probably due to its minimal symptoms [22, 30, 38]. However, we detected many cases with extensive lesions, sometimes affecting multiple body parts. In these cases, children were often affected by more than one fungal disease. These cases may be a reflection of hot climate, household overcrowding, and poor socio-economic status.

We found higher prevalence for fungal infections including pityriasis versicolor and tinea capitis in boys which was also consistent with previous studies [36, 38]. The role of gender in susceptibility to fungal infections is unclear. Yet, this may be due to more closely cropped hair in boys and more intimate contact between them, for instance during playing, thus facilitating transmission. In accordance with previous studies [36], we also found a higher prevalence of tinea capitis in younger children, a condition with a propensity of natural healing with age, whereas older children had higher prevalence of pityriasis versicolor, a superficial fungal infection that is caused by yeasts of the genus *Malassezia* members of which are mostly lipophilic [39]. As secretion of skin lipids increases with age, older children are more prone to this infection whereas the converse is true for susceptibility to tinea capitis.

Allergies including atopic dermatitis have been previously documented to be associated with air pollution [40, 41]. In our study, we found that children with greater exposure to air pollution not only had more inflammatory skin diseases, such as eczema, but also more fungal infections. Air pollutants could enter the skin directly or via hair follicles and sweat/sebaceous glands, thereby damaging the skin barrier function [42]. This could lead to the development of various skin diseases, not just allergies. Studies in this area are still lacking, and our findings are the first to report a possible association between air quality and skin diseases in sub-Saharan Africa.

Although some studies have reported higher rates of skin infections in other parts of the world [18, 29, 43, 44], the prevalences we report (including scabies, bacterial and viral infections) were in the same range as those reported in sub-Saharan Africa by Hogewoning *et al*. [30] Furthermore, these rates may vary significantly by community, and over time, even in resource-limited settings [43]. More studies comparing the rates of skin diseases within the same continent and region would be helpful in the future.

Our study was met with very good community acceptance. The most important reasons for this were: 1) the dermatologists provided diagnoses not only for skin NTDs but for all skin conditions; 2) we provided therapy free of charge for all treatable skin diseases except tinea capitis; and 3) our study was coupled with a mass drug administration campaign for soil-transmitted helminthiasis.

The decision to provide treatment for all survey participants affected by skin disease (except for tinea capitis) was, a difficult one as the high prevalence of skin diseases posed a challenge for our project as the needs for medications and dermatologists exceeded our capacity. This forced us to reduce the number of our study sites in phase 2. However, had we conducted a purely diagnostic survey, our project may not have achieved the same level of community acceptance and parental consent which would have severely hampered the accuracy of our results. Our experience shows that it is important to reach an agreement between all stakeholders about the provision of treatment before the start of a diagnostic survey, and also be logistically and financially prepared. Clear international guidelines for the provision of treatment for similar diagnostic studies are urgently needed for resource-limited settings.

Given the appropriate training, village nurses have the capacity to not only detect but also diagnose simple skin diseases. In our study, they would have been very interested to do so. Studies from Mali and impoverished states of Mexico reported that providing local healthcare providers/workers with a one-day training on skin diseases significantly benefited their patients [45, 46]. Most importantly, widespread reliable access to Internet and mobile phones facilitates tele-dermatology which is another way forward with great potential to compensate for the lack of experts in field [3, 4, 16, 47–49].

There are several limitations to our study. First, we cannot generalize our findings to a larger population as non-probability sampling was used. However, non-probability sampling is justified because we planned to do exploratory studies rather than obtaining results for generalization to the entire country population. Secondly, the financial and logistical constraints led us to reduce the number of our targeted villages (16 to 9). Finally, school skin surveys alone do not reach all children, and therefore, our findings regarding prevalence of skin diseases and detection of skin NTDs only apply to schoolchildren. To overcome this limitation, a combined approach should be considered in detection of skin NTDs, especially for areas with high endemicity. Researchers in Cameroon have done so by successfully combining community-surveys and school-surveys for BU and yaws detection [50].

Despite its limitations and our relative lack of skin NTD detection, we believe that our study provides important epidemiological and logistical information relevant for integrating skin NTD control programs at the local and international levels. In particular, programmes designed to detect early visual clues for the detection of NTDs will inevitably encounter many common skin diseases and should be empowered to manage these simply and effectively.

In conclusion, we carried out the first large-scale integrated, multi-skin NTD survey in Côte d’Ivoire. Our two-phase strategy effectively reached a large population, but the detection of skin NTDs was likely limited because of the relatively low schooling and attendance rate. We found a high prevalence of skin diseases among schoolchildren, possibly due to hot climate, household overcrowding, and poor socio-economic status, but also from air pollution. This requires more attention. With the lessons learned, we currently plan to expand the project to a wider region to further explore its potential to better integrate skin NTD screening in the public health agenda ideally part of the national school health, which would ensure the sustainability of our approach.

## Supporting information

S1 STROBE checklist (PDF)

S1 Informed consent form (PDF)

S1 List Registered primary schools in Adzopé, Côte d’Ivoire 2015–2016. (PDF)

S1 Data (XLSX)
