# Supplementary material for: Skin disease prevalence study in schoolchildren in rural Côte d'Ivoire: Implications for integration of neglected skin diseases (skin NTDs)
Source: PLoS Negl Trop Dis. 2018 May 17;12(5):e0006489. doi: 10.1371/journal.pntd.0006489 (PMC5976208; doi:10.1371/journal.pntd.0006489)
Supplement: S1 STROBE checklist — (PDF) [file pntd.0006489.s001.pdf]

STROBE Statement—checklist of items that should be included in reports of observational studies

	Item No	Recommendation	Page No.	Relevant text from manuscript
**Title and abstract**	1	(*a*) Indicate the study's design with a commonly used term in the title or the abstract	3	Line 9-11
		(*b*) Provide in the abstract an informative and balanced summary of what was done and what was found	3-4	Line 1-31
**Introduction**				
Background/rationale	2	Explain the scientific background and rationale for the investigation being reported	6-8	Line 53-90
Objectives	3	State specific objectives, including any prespecified hypotheses	8	Line 91-95
**Methods**				
Study design	4	Present key elements of study design early in the paper	10	Line 120
Setting	5	Describe the setting, locations, and relevant dates, including periods of recruitment, exposure, follow-up, and data collection	9-13	Line 99-107+119-150
Participants	6	(*a*) *Cohort study*—Give the eligibility criteria, and the sources and methods of selection of participants. Describe methods of follow-up<br>*Case-control study*—Give the eligibility criteria, and the sources and methods of case ascertainment and control selection. Give the rationale for the choice of cases and controls<br>*Cross-sectional study*—Give the eligibility criteria, and the sources and methods of selection of participants	9-10	Line 108-117
		(*b*) *Cohort study*—For matched studies, give matching criteria and number of exposed and unexposed<br>*Case-control study*—For matched studies, give matching criteria and the number of controls per case		
Variables	7	Clearly define all outcomes, exposures, predictors, potential confounders, and effect modifiers. Give diagnostic criteria, if applicable	N/A	
Data sources/ measurement	8*	For each variable of interest, give sources of data and details of methods of assessment (measurement). Describe comparability of assessment methods if there is more than one group	N/A	
Bias	9	Describe any efforts to address potential sources of bias	9-11	Line 112-114, 123-124, 137-138
Study size	10	Explain how the study size was arrived at	9-12	Line 108-116, 141-143
Quantitative variables	11	Explain how quantitative variables were handled in the analyses. If applicable, describe which groupings were chosen and why	12	Line 151-159
Statistical methods	12	(*a*) Describe all statistical methods, including those used to control for confounding	12-13	Line 151, 160-162

		(*b*) Describe any methods used to examine subgroups and interactions		
		(*c*) Explain how missing data were addressed		
		(*d*) *Cohort study*—If applicable, explain how loss to follow-up was addressed<br>*Case-control study*—If applicable, explain how matching of cases and controls was addressed<br>*Cross-sectional study*—If applicable, describe analytical methods taking account of sampling strategy		
		(*e*) Describe any sensitivity analyses		
**Results**				
Participants	13*	(a) Report numbers of individuals at each stage of study—eg numbers potentially eligible, examined for eligibility, confirmed eligible, included in the study, completing follow-up, and analysed	13-14	Line 172-181
		(b) Give reasons for non-participation at each stage	13-14	Line 174, 178-181
		(c) Consider use of a flow diagram	12	Line 148
Descriptive data	14*	(a) Give characteristics of study participants (eg demographic, clinical, social) and information on exposures and potential confounders	14	Line 181-184
		(b) Indicate number of participants with missing data for each variable of interest		
		(c) *Cohort study*—Summarise follow-up time (eg, average and total amount)		
Outcome data	15*	*Cohort study*—Report numbers of outcome events or summary measures over time	N/A	
		Case-control study—Report numbers in each exposure category, or summary measures of exposure		
		Cross-sectional study—Report numbers of outcome events or summary measures		
Main results	16	(*a*) Give unadjusted estimates and, if applicable, confounder-adjusted estimates and their precision (eg, 95% confidence interval). Make clear which confounders were adjusted for and why they were included		
		(*b*) Report category boundaries when continuous variables were categorized		
		(*c*) If relevant, consider translating estimates of relative risk into absolute risk for a meaningful time period		
Other analyses	17	Report other analyses done—eg analyses of subgroups and interactions, and sensitivity analyses	14-18	Line 185-218
**Discussion**				
Key results	18	Summarise key results with reference to study objectives	19-20	Line 238-251
Limitations	19	Discuss limitations of the study, taking into account sources of potential bias or imprecision. Discuss both direction and magnitude of any potential bias	25	Line 331-341

Interpretation	20	Give a cautious overall interpretation of results considering objectives, limitations, multiplicity of analyses, results from similar studies, and other relevant evidence	20-26	Line 252-344
Generalisability	21	Discuss the generalisability (external validity) of the study results	25	Line 331-337

Other information

Funding	22	Give the source of funding and the role of the funders for the present study and, if applicable, for the original study on which the present article is based		This study was supported by the following: 1) Sasakawa Memorial Health Foundation, Japan (http://www.smhf.or.jp/e/); 2) Grant-in-Aid for Scientific Research <KAKENHI>, Japan (https://www.jsps.go.jp/english/e-grants/index.html), grant number: 16K21656; 3) National Center for Global Health and Medicine, Japan (http://www.ncgm.go.jp/100/010/index.html), grant number: 26-120; 4) Ohyama Health Foundation, Japan (http://ohfin.com); 5) St. Luke's Life Science, Japan (https://cce.luke.ac.jp/center/about/index.html); and 6) Research Program on Emerging and Re-emerging Infectious Diseases, Japan Agency for Medical Research and Development (AMED), Japan (http://www.amed.go.jp/en/), grant number: 16fk0108203j0002. The funders had no role in study design, data collection and analysis, decision to publish, or preparation of the manuscript.

*Give information separately for cases and controls in case-control studies and, if applicable, for exposed and unexposed groups in cohort and cross-sectional studies.

Note: An Explanation and Elaboration article discusses each checklist item and gives methodological background and published examples of transparent reporting. The STROBE checklist is best used in conjunction with this article (freely available on the Web sites of PLoS Medicine at http://www.plosmedicine.org/, Annals of Internal Medicine at http://www.annals.org/, and Epidemiology at http://www.epidem.com/). Information on the STROBE Initiative is available at www.strobe-statement.org.
