# Supplementary material for: Skin disease prevalence study in schoolchildren in rural Côte d'Ivoire: Implications for integration of neglected skin diseases (skin NTDs)
Source: PLoS Negl Trop Dis. 2018 May 17;12(5):e0006489. doi: 10.1371/journal.pntd.0006489 (PMC5976208; doi:10.1371/journal.pntd.0006489)
Supplement: S1 Informed consent form — (PDF) [file pntd.0006489.s002.pdf]

Etude de prévalence des maladies de la peau et des phanères chez les enfants d'âge scolaire dans le district sanitaire d'Adzopé

Formulaire de consentement éclairé

TITRE DU PROJET

Etude de prévalence des maladies de la peau chez les enfants d'âge scolaire dans le district sanitaire d'Adzopé

Je soussigné (e) ………………………………………………………………

- certifie avoir été informé(e) sur les objectifs et le déroulement de l'enquête, par l'investigateur chargé de l'étude dont le nom figure au bas de cette page,
- affirme avoir lu attentivement et compris les informations écrites fournies en annexe, informations à propos desquelles j'ai pu poser toutes les questions que je souhaitais,
- certifie avoir été informé(e) des avantages qui sont associés à cette enquête, et des contraintes qu'impliquait ma participation,
- atteste qu'un temps de réflexion suffisant m'a été accordé,
- ai été informé(e) du fait que je pouvais ne pas participer à cette étude sans préjudice d'aucune sorte,
- certifie avoir donné mon accord pour subir des prélèvements d'échantillons,
- consens à ce que les données recueillies pendant l'étude puissent être transmises à des personnes extérieures (notamment les différents partenaires de l'étude), elles-mêmes tenues à respecter la confidentialité de ces informations,

J'accepte donc de participer librement et sans contrainte à l'enquête.

Nom, prénom du patient :

……………………………………………………………

Date et signature: ……. / ………. /……….

Nom, prénom du parent ou tuteur légal

……………………………………………………………

Date et signature: ……… / ………. / ………….

Nom, prénom du témoin impartial

……………………………………………………………

Date et signature :

Nom, prénom du personnel investigateur

……………………………………………………………

Coordonnées de l'investigateur:

Tél : ……………………………………………………

e-mail :

Date et signature : ……. / ………. / ………….

NB : ***Pour les sujets ne sachant pas lire, la présence d'un témoin est exigée***
