# Supplementary material for: Skin disease prevalence study in schoolchildren in rural Côte d'Ivoire: Implications for integration of neglected skin diseases (skin NTDs)
Source: PLoS Negl Trop Dis. 2018 May 17;12(5):e0006489. doi: 10.1371/journal.pntd.0006489 (PMC5976208; doi:10.1371/journal.pntd.0006489)
Supplement: S1 List — (PDF) [file pntd.0006489.s003.pdf]

MINISTERE DE L'EDUCATION NATIONALE
****
DIRECTION REGIONALE D'**ADZOPE**
****
INSPECTION DE L'ENSEIGNEMENT
PRIMAIRE D'**ADZOPE**
****
BP 201 ADZOPE / Tél.: 23-54-02-21
Email : iepadzope1@gmail.com

EFFECTIFS ELEVES PAR CLASSE ET PAR ECOLE PRIMAIRE 2015 - 2016

ECOLES PRIMAIRES PUBLIQUES

N°	ECOLE	CP1			CP2			CE1			CE2			CM1			CM2			TOTAL		
		G	F	T	G	F	T	G	F	T	G	F	T	G	F	T	G	F	T	G	F	T
1	ABOU SEKAKOI	15	10	**25**	08	07	**15**	08	04	**12**	09	10	**19**	10	06	**16**	10	10	**20**	60	47	**107**
2	ADONKOI -1	03	07	**10**	22	24	**46**	14	21	**35**	16	24	**40**	12	16	**28**	10	15	**25**	77	107	**184**
3	ADONKOI -2	03	07	**10**	20	18	**38**			**00**	19	19	**38**	14	09	**23**	09	13	**22**	65	66	**131**
4	AHOKOI	19	08	**27**	21	09	**30**	15	17	**32**	19	11	**30**	13	06	**19**	10	07	**17**	97	58	**155**
5	AHOUABO -1	24	26	**50**	29	34	**63**	35	35	**70**	39	30	**69**	41	29	**70**	32	18	**50**	200	172	**372**
6	AHOUABO -2	24	26	**50**	29	26	**55**	25	21	**46**	28	27	**55**	19	30	**49**	20	24	**44**	145	154	**299**
7	AHOUABO -3	27	23	**50**	24	27	**51**	29	22	**51**	23	23	**46**	27	15	**42**	20	34	**54**	150	144	**294**
8	AHOUABO -4	31	19	**50**	15	23	**38**	13	15	**28**	15	15	**30**	05	16	**21**	13	18	**31**	92	106	**198**
9	ANANGUIE -1	31	29	**60**	28	24	**52**	31	25	**56**	26	19	**45**	32	22	**54**	21	19	**40**	169	138	**307**
10	ANANGUIE -2	30	29	**59**	34	39	**73**	39	27	**66**	32	34	**66**	34	22	**56**	28	23	**51**	197	174	**371**
11	ANANGUIE -3	46	27	**73**	55	47	**102**	38	42	**80**	54	27	**81**	33	33	**66**	23	44	**67**	249	220	**469**
12	ANANGUIE -4	39	25	**64**	34	20	**54**	27	26	**53**	25	27	**52**	22	22	**44**	28	24	**52**	175	144	**319**
13	ANNEPE -1	39	21	**60**	24	29	**53**	31	34	**65**	24	15	**39**	36	29	**65**	21	19	**40**	175	147	**322**
14	ANNEPE -2	18	21	**39**	21	29	**50**	31	27	**58**	22	14	**36**	21	06	**27**	10	07	**17**	123	104	**227**
15	ANNEPE -3	20	28	**48**			**00**	24	16	**40**	11	09	**20**			**00**	12	08	**20**	67	61	**128**
16	APIADJI -1			**00**	21	24	**45**	15	18	**33**	21	30	**51**	26	16	**42**	62	27	**89**	145	115	**260**
17	APIADJI -2	17	10	**27**	16	08	**24**	18	08	**26**	15	21	**36**			**00**			**00**	66	47	**113**
18	ASSAMBROU ADOPO	29	32	**61**	29	32	**61**	23	41	**64**	29	36	**65**	34	35	**69**	30	27	**57**	174	203	**377**
19	ASSIKOI -1	24	31	**55**	30	23	**53**	19	24	**43**	28	26	**54**	20	16	**36**	20	16	**36**	141	136	**277**
20	ASSIKOI -2	29	24	**53**	29	26	**55**	24	30	**54**	30	17	**47**	19	21	**40**	14	18	**32**	145	136	**281**
21	ASSIKOI -3	28	27	**55**	25	20	**45**	25	19	**44**	30	15	**45**	34	31	**65**	21	16	**37**	163	128	**291**

22	BASSADZIN -1	30	20	**50**	35	27	**62**	31	22	**53**	29	19	**48**	24	25	**49**	26	25	**51**	175	138	**313**
23	BASSADZIN -2	23	31	**54**	26	27	**53**	20	23	**43**	21	30	**51**	30	19	**49**	17	21	**38**	137	151	**288**
24	BEKOUKUIFFIN	31	25	**56**	40	29	**69**	41	31	**72**	33	31	**64**	25	37	**62**	24	33	**57**	194	186	**380**
25	BIASSO -1	24	26	**50**	31	33	**64**	27	33	**60**	33	25	**58**	35	25	**60**	30	15	**45**	180	157	**337**
26	BIASSO -2	33	27	**60**	28	34	**62**	30	30	**60**	35	25	**60**	35	30	**65**	34	22	**56**	195	168	**363**
27	BIASSO -3	32	23	**55**	26	24	**50**	25	20	**45**	24	21	**45**	33	26	**59**	18	27	**45**	158	141	**299**
28	BOUAPE -1	22	31	**53**	28	24	**52**	26	30	**56**	22	20	**42**	24	18	**42**	34	40	**74**	156	163	**319**
29	BOUAPE -2	27	19	**46**	21	19	**40**	26	20	**46**	18	24	**42**	30	15	**45**	20	31	**51**	142	128	**270**
30	BOUAPE -3	22	18	**40**	21	19	**40**	29	20	**49**	12	15	**27**	24	12	**36**	10	07	**17**	118	91	**209**
31	CHÂTEAU D'EAU -1	34	38	**72**	42	34	**76**	42	33	**75**	42	37	**79**	40	43	**83**	41	34	**75**	241	219	**460**
32	CHÂTEAU D'EAU -2	35	42	**77**	36	40	**76**	48	35	**83**	37	42	**79**	35	48	**83**	28	45	**73**	219	252	**471**
33	DIASSON -1	30	28	**58**	31	16	**47**	27	34	**61**	22	31	**53**	30	29	**59**	32	26	**58**	172	164	**336**
34	DIASSON -2	35	17	**52**	35	31	**66**	35	33	**68**	33	21	**54**	24	17	**41**	13	17	**30**	175	136	**311**
35	DJOUGBOSSO	16	10	**26**	10	17	**27**	21	19	**40**	06	12	**18**	14	15	**29**	13	14	**27**	80	87	**167**
36	HABITAT -1	32	31	**63**	34	43	**77**	34	47	**81**	38	42	**80**	38	40	**78**	26	42	**68**	202	245	**447**
37	HABITAT -2	30	20	**50**	34	30	**64**	32	30	**62**	28	36	**64**	34	30	**64**	38	30	**68**	196	176	**372**
38	INSTITUT RAOUL FOL.	37	39	**76**	35	29	**64**	26	36	**62**	35	28	**63**	50	25	**75**	36	44	**80**	219	201	**420**
39	KAUDJIS JOSEPH -1	26	26	**52**	26	21	**47**	33	24	**57**	31	24	**55**	20	34	**54**	23	34	**57**	159	163	**322**
40	KAUDJIS JOSEPH -2	31	23	**54**	29	30	**59**	32	31	**63**	34	28	**62**	19	39	**58**	22	36	**58**	167	187	**354**
41	LOBO AKOUDZIN -1	21	21	**42**	30	23	**53**	32	29	**61**	27	13	**40**	18	21	**39**	26	32	**58**	154	139	**293**
42	LOBO AKOUDZIN -2	20	30	**50**	28	20	**48**	17	18	**35**	17	21	**38**	15	18	**33**	37	30	**67**	134	137	**271**
43	LOBO HOPE -1	22	18	**40**	22	18	**40**	27	11	**38**	25	10	**35**	49	25	**74**	34	27	**61**	179	109	**288**
44	LOBO HOPE -2	22	18	**40**	16	23	**39**	26	23	**49**	20	22	**42**	15	13	**28**			**00**	99	99	**198**
45	MAMADOU KONE -1	28	27	**55**	27	28	**55**	33	39	**72**	38	27	**65**	25	25	**50**	26	28	**54**	177	174	**351**
46	MAMADOU KONE -2	24	31	**55**	35	40	**75**	43	40	**83**	45	30	**75**	56	29	**85**	25	35	**60**	228	205	**433**
47	MAMADOU KONE -3	32	23	**55**	23	23	**46**	32	27	**59**	34	28	**62**	16	16	**32**	28	28	**56**	165	145	**310**
48	MAMADOU KONE -4			**00**			**00**			**00**			**00**	17	17	**34**	28	28	**56**	45	45	**90**
49	MASSANDJI	21	22	**43**	16	32	**48**	10	17	**27**	22	13	**35**	15	24	**39**	14	18	**32**	98	126	**224**
50	MOAPE -1	23	25	**48**	28	26	**54**	24	23	**47**	27	34	**61**	28	36	**64**	24	31	**55**	154	175	**329**
51	MOAPE -2	31	22	**53**	32	22	**54**	28	39	**67**	31	34	**65**	36	41	**77**	33	27	**60**	191	185	**376**
52	MOAPE -3	30	16	**46**	26	21	**47**	25	23	**48**	30	26	**56**	40	30	**70**	34	19	**53**	185	135	**320**
53	MOAPE -4	26	21	**47**	28	29	**57**	21	13	**34**			**00**			**00**			**00**	75	63	**138**

54	N'KOUPE -1	34	30	**64**	30	23	**53**	19	27	**46**	26	28	**54**	21	33	**54**	24	24	**48**	154	165	**319**
55	N'KOUPE -2	28	29	**57**	35	27	**62**	30	27	**57**	32	25	**57**	41	26	**67**	30	28	**58**	196	162	**358**
56	NYAN -1	21	21	**42**	26	24	**50**	17	23	**40**	23	22	**45**	23	22	**45**	28	32	**60**	138	144	**282**
57	NYAN -2	13	37	**50**	05	38	**43**	06	29	**35**	07	33	**40**	05	24	**29**	22	25	**47**	58	186	**244**
58	P.E.I. AHOUABO			**00**		02	**02**			**00**			**00**	22	29	**51**	13	22	**35**	35	53	**88**
59	P.E.I. ANANGUIE	20	25	**45**	27	21	**48**	27	19	**46**	26	22	**48**	31	22	**53**	25	20	**45**	156	129	**285**
60	P.E.I. BOUAPE	09	06	**15**			**00**			**00**			**00**			**00**			**00**	09	06	**15**
61	P.E.I. MOAPE	12	23	**35**	24	16	**40**	22	13	**35**	21	16	**37**	11	09	**20**	10	12	**22**	100	89	**189**
62	PLATEAU -1	41	29	**70**	31	40	**71**	37	34	**71**	38	28	**66**	32	18	**50**	33	17	**50**	212	166	**378**
63	PLATEAU -2	23	42	**65**	30	32	**62**	27	23	**50**	30	30	**60**	22	16	**38**	19	15	**34**	151	158	**309**
64	POSTE -1	27	23	**50**	25	25	**50**	34	22	**56**	25	32	**57**	31	27	**58**	26	31	**57**	168	160	**328**
65	POSTE -2	31	24	**55**	23	27	**50**	26	24	**50**	24	29	**53**	29	24	**53**	29	35	**64**	162	163	**325**
66	QUARTIER LYCEE -1	38	35	**73**	48	34	**82**	43	37	**80**	42	33	**75**	41	39	**80**	24	36	**60**	236	214	**450**
67	QUARTIER LYCEE -2	28	41	**69**	42	36	**78**	40	35	**75**	32	46	**78**	33	42	**75**	37	33	**70**	212	233	**445**
68	METHODISTE	20	30	**50**	19	15	**34**	19	15	**34**	20	22	**42**	31	26	**57**	15	10	**25**	124	118	**242**
69	NOTRE DAME -1	21	21	**42**	13	23	**36**	14	14	**28**	16	19	**35**	16	26	**42**	17	18	**35**	97	121	**218**
70	NOTRE DAME -2	14	18	**32**	21	18	**39**	15	14	**29**	18	18	**36**	14	22	**36**	12	16	**28**	94	106	**200**
71	PERE MIET	25	12	**37**	27	18	**45**	22	18	**40**			**00**			**00**			**00**	74	48	**122**
72	SAINT CHARLES B.	14	16	**30**	15	12	**27**	18	18	**36**	23	25	**48**	30	26	**56**	23	28	**51**	123	125	**248**
73	BISMA			**00**			**00**			**00**			**00**			**00**			**00**	00	00	**00**
74	DAR AL HADISS	31	36	**67**	28	47	**75**	27	37	**64**	34	31	**65**	22	14	**36**	32	14	**46**	174	179	**353**
75	IMAM HOUSSEINE	19	38	**57**	31	20	**51**	20	20	**40**	16	11	**27**	11	10	**21**	18	08	**26**	115	107	**222**
76	SEKOU OUMAR CISSE	20	10	**30**	25	26	**51**	15	20	**35**	17	13	**30**	11	10	**21**	12	08	**20**	100	87	**187**
77	EPP AMAKPÉ TABOA -1	27	21	**48**	30	26	**56**	26	30	**56**	22	33	**55**	27	27	**54**	27	21	**48**	159	158	**317**
78	EPP AMAKPÉ TABOA -2	22	21	**43**	19	38	**57**	27	27	**54**	26	31	**57**	26	26	**52**	31	21	**52**	151	164	**315**
79	EPP SAMHAT ABDULATIF	16	19	**35**	27	16	**43**	24	20	**44**	18	18	**36**	19	14	**33**	16	29	**45**	120	116	**236**
80	EPP ADZOPÉ TP -1	24	17	**41**	22	14	**36**	20	24	**44**	26	16	**42**	19	24	**43**	22	16	**38**	133	111	**244**
81	EPP ADZOPÉ TP -2	16	22	**38**	17	18	**35**	18	20	**38**	22	22	**44**	26	19	**45**	15	26	**41**	114	127	**241**
82	EPP ADZOPÉ TP -3	23	22	**45**	20	23	**43**	28	31	**59**	35	21	**56**	13	23	**36**	22	27	**49**	141	147	**288**
83	EPP OKN	19	23	**42**	16	19	**35**	25	31	**56**	15	18	**33**	19	16	**35**	10	11	**21**	104	118	**222**
84	EPP MIADZIN -1	18	19	**37**	22	17	**39**	20	17	**37**	13	19	**32**	08	13	**21**	10	08	**18**	91	93	**184**
85	EPP MIADZIN -2	12	17	**29**	19	13	**32**	18	17	**35**	18	14	**32**	17	11	**28**	11	12	**23**	95	84	**179**

86	EPP ZODJI	07	08	**15**	04	09	**13**	10	09	**19**	07	07	**14**	11	02	**13**	06	09	**15**	45	44	**89**
87	EPP DIAPÉ -1	29	08	**37**	23	20	**43**	24	17	**41**	25	14	**39**	21	21	**42**	22	18	**40**	144	98	**242**
88	EPP DIAPÉ -2	18	25	**43**	21	22	**43**	31	25	**56**	22	17	**39**	17	12	**29**	16	09	**25**	125	110	**235**
89	EPP DIAPÉ -3	21	19	**40**	25	18	**43**	21	19	**40**	22	20	**42**	21	14	**35**	24	13	**37**	134	103	**237**
90	EPP DIAPÉ -4	13	14	**27**	15	19	**34**	32	17	**49**	08	15	**23**	16	12	**28**	21	07	**28**	105	84	**189**
91	EPP AGOU -1	23	27	**50**	30	25	**55**	25	26	**51**	20	25	**45**	19	27	**46**	21	21	**42**	138	151	**289**
92	EPP AGOU -2	23	27	**50**	39	29	**68**	21	26	**47**	29	24	**53**	19	18	**37**	19	15	**34**	150	139	**289**
93	EPP AGOU -3	18	25	**43**	25	23	**48**	28	17	**45**	17	18	**35**	13	32	**45**	19	12	**31**	120	127	**247**
94	EPP N'GUESSANKOI	08	19	**27**	12	18	**30**	10	06	**16**	21	24	**45**	13	14	**27**	18	17	**35**	82	98	**180**
95	EPP AYALO	12	17	**29**	26	14	**40**	22	17	**39**	11	17	**28**	06	11	**17**	16	13	**29**	93	89	**182**
96	EPP ABIÉ NORD	25	25	**50**	33	21	**54**	25	24	**49**	25	24	**49**	22	20	**42**	15	26	**41**	145	140	**285**
97	EPP ABIÉ EST	28	24	**52**	11	20	**31**	18	12	**30**	21	21	**42**	11	17	**28**	22	20	**42**	111	114	**225**
98	EPP ABIÉ SUD A	31	22	**53**	24	21	**45**	23	21	**44**	18	17	**35**	23	23	**46**	18	16	**34**	137	120	**257**
99	EPP ABIÉ SUD B	19	24	**43**	28	22	**50**	18	13	**31**	18	16	**34**	23	12	**35**	20	17	**37**	126	104	**230**
100	EPP YAKASSÉ MÉ -1	42	27	**69**	42	37	**79**	33	34	**67**	42	42	**84**	42	36	**78**	39	30	**69**	240	206	**446**
101	EPP YAKASSÉ MÉ -2	35	33	**68**	37	28	**65**	38	33	**71**	41	33	**74**	36	32	**68**	29	28	**57**	216	187	**403**
102	EPP YAKASSÉ MÉ -3	37	35	**72**	36	26	**62**	36	30	**66**	42	33	**75**	45	30	**75**	26	21	**47**	222	175	**397**
103	EPP YAKASSÉ MÉ -4	44	30	**74**	40	32	**72**	36	26	**62**			**00**			**00**			**00**	120	88	**208**
104	EPP BÉCÉDI BRIGNAN -1	32	34	**66**	37	30	**67**	33	32	**65**	38	28	**66**	35	30	**65**	32	30	**62**	207	184	**391**
105	EPP BÉCÉDI BRIGNAN -2	29	39	**68**	38	30	**68**	30	34	**64**	31	36	**67**	31	29	**60**	32	26	**58**	191	194	**385**
106	EPP BÉCÉDI BRIGNAN -3	30	27	**57**	47	17	**64**	30	32	**62**	31	28	**59**	27	26	**53**	41	26	**67**	206	156	**362**
107	EPP BÉCÉDI BRIGNAN -4	31	18	**49**	29	20	**49**	31	18	**49**	28	22	**50**	28	21	**49**	27	22	**49**	174	121	**295**
108	EPP BÉCÉDI BRIGNAN -5			**00**	19	16	**35**			**00**	13	10	**23**			**00**	26	25	**51**	58	51	**109**
109	EPP BÉCÉDI BRIGNAN -6	32	28	**60**	37	28	**65**	28	24	**52**	28	25	**53**	30	29	**59**	48	13	**61**	203	147	**350**
110	PEI BÉCÉDI BRIGNAN	14	11	**25**	20	26	**46**	23	12	**35**	24	19	**43**			**00**			**00**	81	68	**149**
111	EPP MAFA MAFOU	13	14	**27**	10	11	**21**	14	06	**20**	08	03	**11**	10	07	**17**	10	09	**19**	65	50	**115**
112	EPP MOPÉ	04	06	**10**	10	10	**20**	03	10	**13**	10	07	**17**	04	07	**11**	17	04	**21**	48	44	**92**
113	EPP BÉCÉDI ANON -1	21	23	**44**	18	14	**32**	16	21	**37**	20	28	**48**	15	21	**36**	27	19	**46**	117	126	**243**
114	EPP BÉCÉDI ANON -2	17	25	**42**	19	18	**37**	20	26	**46**	15	15	**30**	18	18	**36**	19	19	**38**	108	121	**229**
115	EPP BOUDÉPÉ -1	20	20	**40**	16	21	**37**	21	16	**37**	25	18	**43**	27	12	**39**	26	16	**42**	135	103	**238**
116	EPP BOUDÉPÉ -2	17	13	**30**	13	21	**34**	15	23	**38**	14	23	**37**	14	10	**24**	13	17	**30**	86	107	**193**
117	EPP BOUDÉPÉ -3	20	20	**40**	16	21	**37**	21	16	**37**	25	18	**43**	27	12	**39**	26	16	**42**	135	103	**238**

118	EPP AKOUDZIN -1	11	15	**26**	25	24	**49**	18	14	**32**	12	12	**24**	21	06	**27**	11	11	**22**	98	82	**180**
119	EPP AKOUDZIN -2	16	24	**40**	18	22	**40**	26	14	**40**	13	22	**35**	17	16	**33**	15	12	**27**	105	110	**215**
120	EPP AKOUDZIN -3	13	12	**25**	17	15	**32**	28	12	**40**	09	16	**25**	13	11	**24**	24	12	**36**	104	78	**182**
121	EPP AKOUDZIN -4	18	21	**39**			**00**			**00**	11	25	**36**			**00**	12	18	**30**	41	64	**105**
122	EPP ANDÉ -1	22	21	**43**	29	11	**40**	19	16	**35**	19	15	**34**	19	16	**35**	16	22	**38**	124	101	**225**
123	EPP ANDÉ -2	27	20	**47**	29	19	**48**	25	19	**44**	31	25	**56**	12	18	**30**	24	17	**41**	148	118	**266**
124	EPC YAKASSÉ MÉ	23	25	**48**	35	30	**65**	25	28	**53**	30	26	**56**	33	22	**55**	25	10	**35**	171	141	**312**
125	EPM YAKASSÉ MÉ	23	31	**54**	25	21	**46**	23	22	**45**	24	28	**52**	28	29	**57**	17	16	**33**	140	147	**287**
TOTAL		**2878**	**2781**	**5659**	**3088**	**2858**	**5946**	**2971**	**2778**	**5749**	**2877**	**2709**	**5586**	**2764**	**2513**	**5277**	**2660**	**2488**	**5148**	**17238**	**16127**	**33365**

NOTES:

* Population of schooling-age children in Adzopé (5-14 years): 52,941 ; Number of children going to primary school: 39,691

** Not all primary shools in Adzopé are included on this list.
